# Evaluating the Behavioural Responses of Healthy Newborn Calves to a Thoracic Squeeze

**DOI:** 10.3390/ani12070840

**Published:** 2022-03-26

**Authors:** Sophia E. Holdsworth, Nikki J. Kells, Emilie Vallée, Neil Ward, David J. Mellor, Ngaio J. Beausoleil

**Affiliations:** 1 Animal Welfare Science and Bioethics Centre, School of Veterinary Science, Massey University, Private Bag 11-222, Palmerston North 4442, New Zealand; s.holdsworth@massey.ac.nz (S.E.H.); n.j.kells@massey.ac.nz (N.J.K.); n.ward@massey.ac.nz (N.W.); d.j.mellor@massey.ac.nz (D.J.M.); 2 EpiCentre, School of Veterinary Science, Private Bag 11-222, Palmerston North 4442, New Zealand; e.vallee@massey.ac.nz

**Keywords:** thoracic squeeze, low vigour, chest squeeze, reflex responses, loss of posture, cessation of movement, neonatal calves, tonic immobility

## Abstract

**Simple Summary:**

Reports of calves born via caesarean section behaving abnormally have led to the application of a therapy called a ‘thoracic squeeze’ that has been used to ‘recover’ low-vigour neonates of other farmed mammal species. The squeeze involves looping a rope around the chest of the animal and pulling it taut, causing a state of reduced responsiveness. Once the squeeze is removed, low-vigour neonates are reported to immediately stand up and display normal behaviours. We aimed to characterise the behavioural responses of healthy newborn calves to a thoracic squeeze using two methods: a rope and an inflation cuff. In total, 13 of the 16 calves squeezed were induced into a state of reduced responsiveness, though their pedal and palpebral reflexes remained present in nearly all of the calves. For nearly half of the calves induced, the squeeze was discontinued before the end of the 10-min period due to spontaneous arousal or abnormal changes in their physiological status. The calves squeezed with the cuff appeared to lose posture and stop moving faster than the calves squeezed with the rope. This study demonstrates that healthy calves born without assistance respond similarly to the squeeze to other mammalian species, and it provides a foundation for the exploration of the mechanisms underlying these responses.

**Abstract:**

A thoracic squeeze has been observed to cause low-vigour neonates of various farmed mammal species, including calves, to enter a state of reduced responsiveness. The removal of the squeeze causes rapid recovery and the expression of normal, healthy behaviours. However, the responses of healthy calves to a thoracic squeeze have not yet been characterized. The responses of 16 healthy newborn calves to a thoracic squeeze are described, along with the effect of the squeeze’s application method on the response. Calves aged between 12 and 36 h were subjected to the squeeze using a rope (*n* = 8) or an inflation cuff (*n* = 8). In total, 13 of the 16 calves were induced into a state of reduced responsiveness, though neural reflexes persisted in nearly all of them. The squeeze was discontinued for nearly half of those induced before the end of the 10-min period, either due to spontaneous arousal or physiological instability. Both methods of application were equally effective at inducing reduced responsiveness, though responses to the cuff appeared to be more rapid than those to the rope. These findings support previous research on piglets and foals, and suggest that the behavioural responses to a thoracic squeeze are generalised across neonates of precocial farmed mammals; the findings provide a foundation for further research exploring the mechanisms underlying the response and the benefits that its application may bring for the performance of husbandry procedures.

## 1. Introduction

The survival of newborn mammals after birth relies on the rapid expression of a particular pattern of behaviours [1]. These behaviours include standing, walking, teat seeking, and sucking colostrum and milk [2]. Any impairment to the expression of these survival-related behaviours soon after birth is characterized as ‘low vigour’ [2]. Low-vigour neonates exhibit abnormalities in standing and walking, a lack of dam-seeking or teat-seeking behaviours, and the lack of a sucking reflex [3]. In some cases, low vigour leads to hypothermia and starvation [4]. Without rapid veterinary intervention, these neonates likely experience welfare compromise, and often die [3,5]. 

Low vigour contributes significantly to mortality rates, with the annual global mortality rate being as high as 15% in calves [6,7]. Higher rates of low vigour have been observed after difficult calving or assisted deliveries [8]. Dystocia rates have been steadily increasing in cow pregnancies, leading to prolonged and assisted births that often result in the calf experiencing hypoxia-induced low vigour after birth [9]. One treatment or prevention option is to perform a caesarean section (C-section) in order to safeguard the health of the cow and calf. The C-section was first performed in Belgium as an emergency intervention to save the calf and cow when dystocia was apparent, but it has since become a routine procedure for large cattle breeds such as Belgian blue breeds, which are growing in popularity globally [10]. However, it is now thought that C-sections may also contribute to the prevalence of low vigour in calves [11].

It has recently been proposed that calves delivered via C-section do not receive adequate body compression because they do not pass through the birth canal [11]. It has been suggested that this compression facilitates the transition from unconsciousness in utero to consciousness after birth [12]. Without this compression, it is theorised that this transition is impaired, which may lead to a delay in the expression of survival behaviours, which is reflected in low vigour [11]. While there is no direct evidence to support this theory, it has formed the basis of a recent therapy for low-vigour neonates. 

The “thoracic squeeze” technique is a proposed therapy for low-vigour newborns that has been considered successful in a number of different mammalian species: foals, calves and lambs [3,11,13]. The thoracic squeeze was recently used on two C-section delivered calves that showed behaviours consistent with low vigour [11]. The application of a firm squeeze to the chest using a soft fabric rope caused these newborns to rapidly enter a ‘sleep-like’ state, characterised by lateral recumbency and the cessation of movements for as long as the squeeze was held [11]. The removal of the squeeze resulted in a rapid expression of healthy survival-related behaviours [11]. 

In order to assess whether this therapy is safe for low-vigour calves, it is first necessary to demonstrate that the squeeze has no adverse effects on healthy calves. To date, there have been no systematic studies evaluating the effects of the thoracic squeeze on healthy calves. As such, the main aim of this study was to describe the responses of healthy newborn calves to a standardised application of the thoracic squeeze. A secondary aim was to evaluate the effect of the method of squeeze application on calves’ responses: a soft fabric rope or a modified inflation cuff.

## 2. Materials and Methods 

The study was undertaken on a commercial dairy farm in the Manawatū region of New Zealand. All of the procedures were approved by the Massey University Animal Ethics Committee (MUAEC Protocol 19/06). 

### 2.1. Selection of the Animals

In accordance with normal farm practices, potential study subjects were removed from the dam between 2 and 24 h of age and placed into a pen in the calf shed with dry woodchip bedding and shelter from the elements for the study. They were assessed for inclusion in the study at 12 to 36 h old. In total, sixteen healthy calves (6 Jersey, 10 Friesian crossbreed) were selected based on the following inclusion criteria (as described in detail by Holdsworth et al. [14]): normal locomotion, sucking reflex, body posture, respiratory rate, heart rate, and rectal temperature. In order to assess locomotion, the calves were encouraged to stand through touch or by manually assisting the calf to its feet, and their movement around the pen was observed. Calves were only included if they could move freely without assistance. The presence of a sucking reflex was tested by feeding the calves approximately 200 mL warm colostrum or cold milk in a bottle. Whether the calves had received colostrum from the dam before being removed, received colostrum or milk replacer in calf feeders, or were unfed before the assessment was unknown. As some calves may not yet have sucked from their dams, a weak sucking reflex was considered an acceptable standard for inclusion. Normal body posture required the head to be at the level of the body, and not pulled back and up while the calf was in sternal recumbency or standing on all four feet. The normal rectal temperature for calves was considered to be between 38 and 39 degrees Celsius [15,16].

### 2.2. Procedures

Data were collected on 8 days over a two-week period. The number of calves tested per day depended on the availability of calves within the desired age range; up to three calves were used in a single study day. The calves were alternately assigned to either the inflation cuff or rope treatment. Overall, eight calves were squeezed using the rope treatment and eight were squeezed using the cuff. Each animal was squeezed only once in order to prevent a cumulative response. Once it was assessed as suitable for inclusion, the test calf was moved from the group calving pen into an adjacent pen for testing. Following treatment and monitoring for complete recovery, the calf was returned to the group pen. All of the procedures were undertaken in the pen whilst maintaining normal industry conditions for lighting, noise and temperature.

For the application of the squeeze, the calf was held in a standing position, before being allowed to lie in sternal recumbency after the start of the squeeze application. The calf was held by a handler with one arm supporting the hind end and one arm around the front of the chest to keep the calf in a standing position. The squeeze device was firmly secured around the thorax, and the squeeze was applied as described by Holdsworth et al. [14]. A timer was started upon the application of the squeeze, and the latency to induction into a state of reduced responsiveness was recorded by identifying two behaviours: lateral recumbency and the cessation of limb movements. The squeeze was applied until the calf met the discontinuation criteria (see Section 2.4 below) or the maximum time of 10 min was reached. Prior research in pigs has demonstrated that 10 min is sufficient to demonstrate squeeze efficacy [14]. Furthermore, this is shorter than the average duration of stage II labour in cows (38–114 min [17,18,19]), ensuring that any risks associated with prolonged squeezing were mitigated. After the squeeze was completed or discontinued, each calf was monitored for 5 min in order to ensure that they had recovered from the squeeze and were displaying the behaviours described in the inclusion criteria [14]. The calves were encouraged into sternal recumbency, and were allowed to remain there for two minutes before being encouraged to stand by a researcher in order to assess their recovery.

### 2.3. Treatments

**Figure 1:**
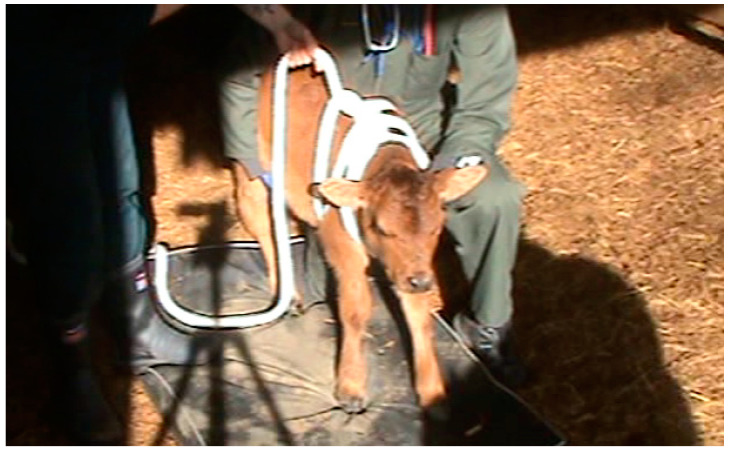
Rope squeeze placed around the thorax of a calf before pulling the rope tight.

The rope squeeze treatment was applied using a soft polyester fabric rope of 3 cm in diameter and 5 m in length. A small loop was already sewn into one end of the rope, which acted as a bowline knot. The rope squeeze was applied as described by Holdsworth et al. [14] (Figure 1).

**Figure 2:**
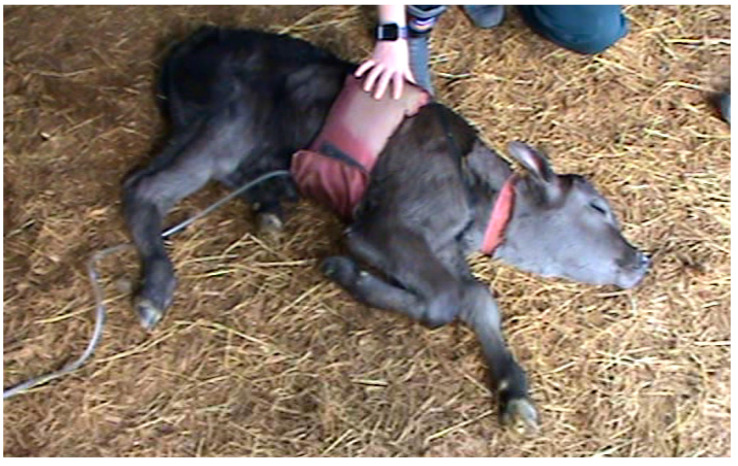
Novel inflation cuff applied to a calf, secured around the thorax with Velcro and fastened to a soft fabric black strap around the neck to prevent slippage onto the abdomen.

The inflation cuff treatment was applied using a custom-designed inflatable band that was wrapped around the thorax and secured snugly using Velcro. The nylon band was 17 cm wide × 97 cm long, and housed an inflatable rubber bladder. These dimensions were determined by measuring the thoracic girth and length of five non-subject calves within the 12–36-h age range. The cuff was applied as described by Holdsworth et al. [14] (Figure 2). Once the cuff was in place, the bladder was inflated to a minimum effective pressure of 110 mm Hg using a compressed air tank with a regulator, with an attached pressure gauge.

### 2.4. Criteria for the Discontinuation of the Squeeze

The discontinuation criteria for the calves during this study were based on criteria developed by Holdsworth et al. [14]. Several physiological variables were evaluated at 2 and 8 min after the start of the squeeze application: heart rate, respiratory rate, oxygen saturation (where possible), oral mucosa colour, rectal temperature, and muscle tone. For more detail on the physiological variables, see Holdsworth et al. [14]. Monitoring these variables ensured that calves remained healthy during the squeeze. When any parameter approached the bounds of the normal range at the 2-min check [16,20], the calves were monitored more frequently. Continuing changes in any of these parameters on subsequent checks triggered the immediate discontinuation of the squeeze. The calves were also monitored for any persistent struggling once the squeeze was applied and during the initial increase in pressure. If the struggling persisted for 2 min after the start of squeeze application, the treatment was discontinued. Likewise, if the calf was calm during the application of the treatment and the initial increase in pressure, but there was a failure of induction by 3 min—that is, no loss of posture or cessation of movement occurred—then the squeeze was immediately discontinued. The squeeze was also discontinued if the calf showed higher intensity arousal (Table 1) before the end of the 10-min period.

### 2.5. Data Collection

Behavioural responses and neural reflex activity following the application of the squeeze were assessed (Table 1), both in real time and by reference to video recordings. The video was captured using one Sony Handycam DCR-SR85 (Sony Corporation, Tokyo, Japan) placed on a tripod with a full view of the whole calf (see Figure 1 and Figure 2 for screenshots of the camera footage), such that subtle behavioural changes such as eye closure could be observed post hoc.

### 2.6. Data Analysis

The aim of this study was to characterise the responses of calves to the thoracic squeeze. Therefore, the data analysis is descriptive in nature. A four-point scale, as described by Holdsworth et al. [14], was used to categorise the responses of individual calves to the squeeze. This was based on whether or not the squeeze successfully induced a state of reduced responsiveness—characterised by a loss of posture and a cessation of movement—and how long this was maintained for.

A: No induction into a state of reduced responsiveness.

B: Induction into a state of reduced responsiveness occurred within 180 s of the start of the application of the squeeze but was not maintained for the full 10-min observation period due to high-intensity arousals.

C: Induction into a state of reduced responsiveness occurred within 180 s of the start of the application of the squeeze, and was maintained for the 10-min squeeze duration.

D: Induction into a state of reduced responsiveness occurred within 180 s of the start of the application of the squeeze, but the squeeze was discontinued due to concerns based on physiological monitoring.

While previous research in piglets included eye closure as an indicator of induction into a state of reduced responsiveness [14], eye closure was removed from the induction criteria due to no calves in this study closing their eyes.

The numbers and percentages (with 95% confidence intervals, calculated using the exact binomial method) of animals in each category are presented. The effect of the induction categories or the method of squeeze application on the time from latency to induction (the loss of posture and the cessation of movement) were explored using Kaplan–Meier graphs. The effect of induction categories or the method of squeeze application on the presence/absence of pedal and palpebral reflexes and the frequency of low-intensity arousals during the squeeze were explored graphically. The rate of low-intensity arousals for the Category B and C calves could only be compared for the time blocks during which calves in both groups were still being squeezed, i.e., up to 6 min after the application of the squeeze. The rate (defined as the number of arousals divided by the total number of minutes from induction to the end of the 2-min block), rather than the actual number of arousals, was compared because the period during which the calves could arouse in the first 2-min block varied depending on how long induction took. The second and third blocks were two minutes long for all of the calves (2 to 4 min and 4 to 6 min after the start of the squeeze, respectively). The averages and standard deviations for each time block were then graphed in order to compare the induction categories and methods of squeeze application. All of the summary statistics and graphical representations were produced using R Studio version 1.2.1335 [23].

## 3. Results

### 3.1. Success and Maintenance of Induction

Thirteen of the sixteen calves squeezed were successfully induced based on a loss of posture and the cessation of movement for 3 s. There were three Category A calves (19%) that were not successfully induced (Table 2. Overall, six out of 16 calves did not complete the 10-min squeeze period (Categories B and D), and 7 did (Category C). The discontinuation of the squeeze due to high-intensity arousals (Category B) occurred consistently between 360 and 510 s, whereas the discontinuation of the squeeze due to physiological instability (Category D) occurred before 360 s.

In the rope group, the squeeze was discontinued for two out of eight calves; both were due to high-intensity arousals at 411 and 510 s. In the cuff group, the squeeze was discontinued for four out of eight calves. Two were discontinued due to a rapid decline in their physiological parameters at 322 and 332 s, and the other two calves were discontinued due to high-intensity arousals at 351 and 464 s.

### 3.2. Relationship between the Induction Category, Time to Induction, and Behaviour during the Squeeze

**Figure 3:**
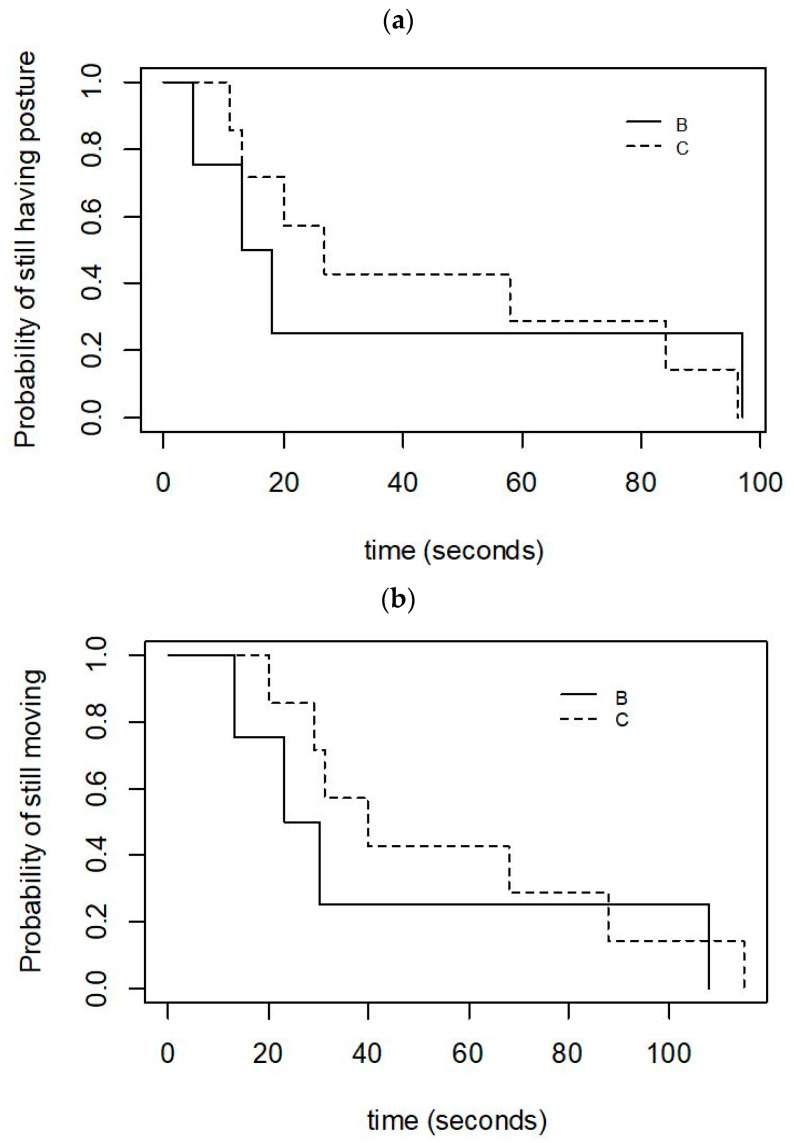
Kaplan–Meier graphs of (**a**) the latency to lose posture, and (**b**) the latency to cease moving in 11 calves that were successfully induced into a state of reduced responsiveness with either the cuff or the rope, and that did not have the squeeze discontinued for health reasons. Category C calves (*n* = 7) maintained a state of reduced responsiveness for the full 10-min squeeze period, whereas the squeeze was discontinued in Category B calves (*n* = 4).

The calves for which the squeeze was discontinued due to high-intensity arousals (Category B) initially lost posture as quickly as the calves that maintained reduced responsiveness for the full 10 min (Category C) (Cat B: Med = 15.5 s, Range = 5–97; Cat C: Med = 27 s, Range = 11–96; Figure 3a). Likewise, the Category B calves appeared to cease moving sooner after the application of the squeeze than did the Category C calves (Cat B: Med = 26.5 s, Range = 13–108; Cat C: Med = 40 s, Range = 20–115; Figure 3b).

**Figure 4:**
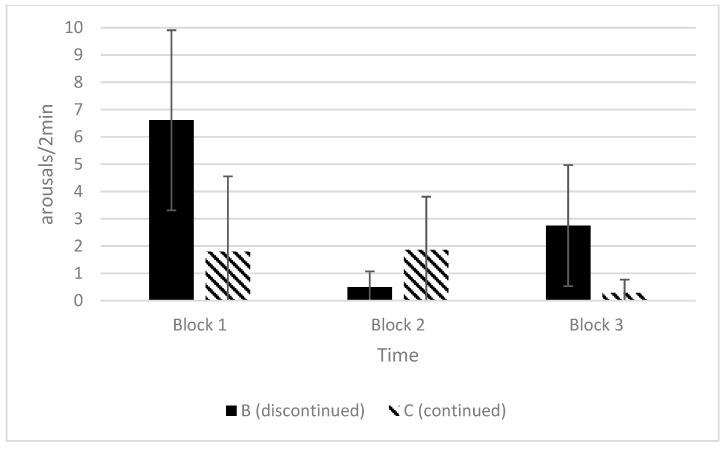
Average (±SD) rate of low-intensity arousals per 2 min calculated for the first three time blocks in the calves for which the squeezed was maintained for the full 10-min period (Category C, *n* = 7) and the calves for which the squeeze was discontinued due to high-intensity arousal (Category B, *n* = 4). The duration of the first time block varied among the individual calves depending on how long it took for the calf to be induced and, as a result, was less than 2 min for some individuals. Blocks 2 and 3 were both two minutes long in all of the calves.

The rate of low-intensity arousals of Category B and C calves could only be compared for the time blocks during which calves in both groups were still being observed (up to 6 min). The calves for which the squeeze was subsequently discontinued (Category B) showed numerically more low-intensity arousals with greater individual variability in blocks 1 and 3, but showed fewer arousals in block 2 compared to the calves that were maintained in a state of reduced responsiveness for the full 10 min (Category C) (Figure 4).

### 3.3. Effect of the Method of Application on the Induction, Discontinuation, and Behaviour during the Squeeze

**Figure 5:**
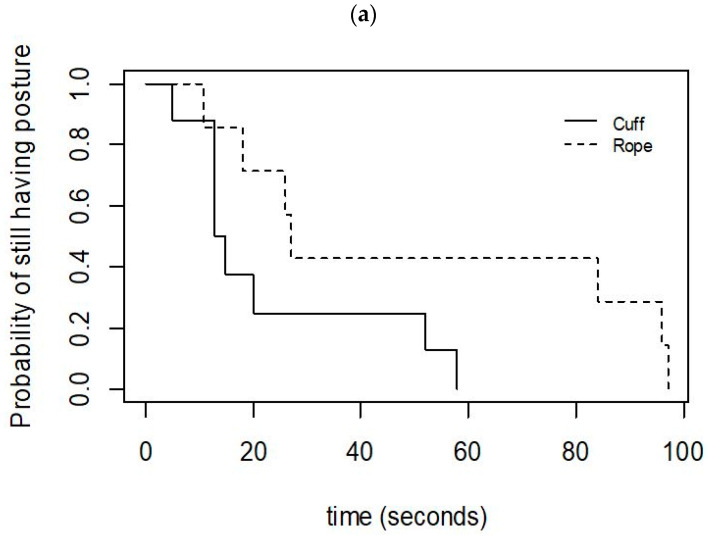

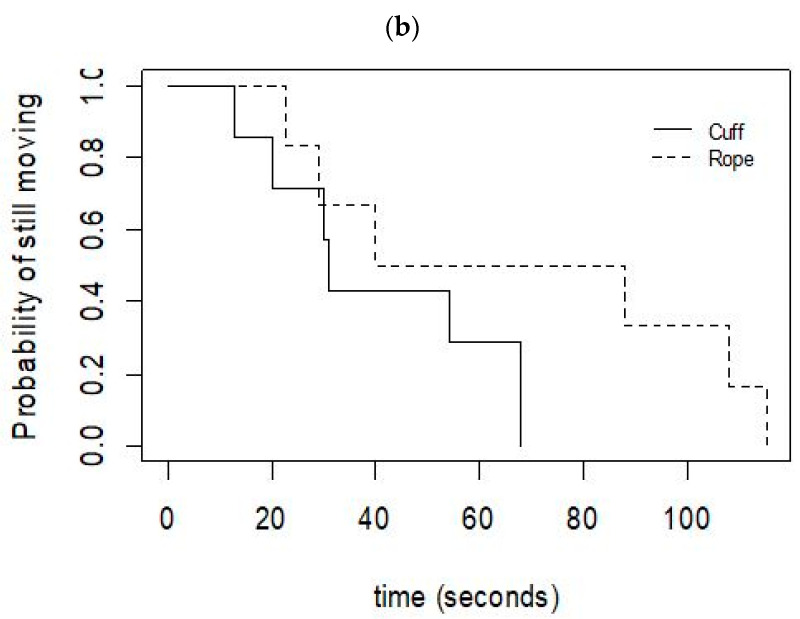
Kaplan–Meier graphs of (**a**) the latency to lose posture, and (**b**) the latency to cease moving in 16 calves that were successfully induced into a state of reduced responsiveness with either the cuff (*n* = 8) or the rope (*n* = 8).

The time to the loss of posture appeared to be shorter in calves squeezed with the cuff than it was in those squeezed with the rope, and the time to the loss of posture was less variable with the cuff (Cuff: Med = 14 s, Range = 5–58 s; Rope: Med = 27 s, Range = 11–97 s; Figure 5a). The calves squeezed with the cuff appeared to stop moving sooner than those squeezed with the rope (Cuff: Med = 31 s, Range = 13–68 s; Rope: Med = 54 s, Range = 23–115 s; Figure 5b).

**Figure 6:**
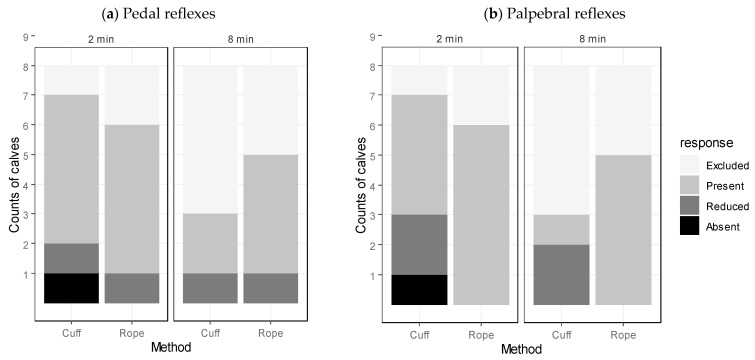
Comparison of (**a**) the pedal reflex responses, and (**b**) the palpebral reflex responses at 2 min and 8 min into the thoracic squeeze for each method of application. “Excluded” refers to calves that did not have their reflexes tested for logistical reasons, or because the squeeze had already been discontinued. “Present” refers to a full limb withdrawal; “Reduced” refers to a slight limb withdrawal; “Absent” refers to a lack of limb responses to reflex testing.

Among the calves for which the squeeze was discontinued due to physiological instability (Category D) or arousal (Category B), those squeezed with the cuff appeared to be discontinued sooner than those squeezed with the rope (Cuff: Med = 341.5 s, Range = 332–464 s, *n* = 4; Rope: Med = 460.5 s, Range = 411–510 s, *n* = 2). At the 2-min reflex testing, one calf squeezed with the cuff and two calves squeezed with the rope could not be tested because they failed to induce (Category A). Of the seven calves tested in the cuff group at this time, six showed a present pedal response, and four showed a present palpebral response. In the rope group, all six calves tested showed present pedal and palpebral responses (Figure 6a,b).

At the 8-min reflex testing, eight calves could not be tested because the squeeze had already been discontinued. In the cuff group, two of the three calves tested showed present pedal and palpebral responses, and one was reduced. In the rope group, four of the five calves tested had a present pedal response, while the one remaining calf showed a reduced response. All of the calves in the rope group showed present palpebral reflex responses (Figure 6a,b).

**Figure 7:**
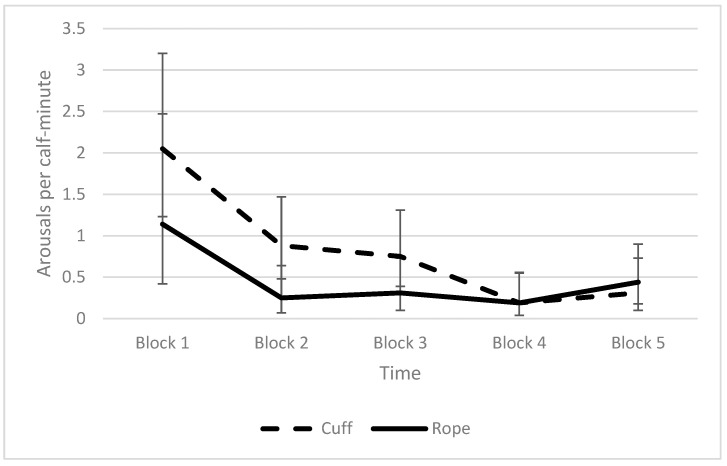
Average (+SD) rate of low-intensity arousals per 2 min with 95% confidence intervals for the calves squeezed with the cuff or rope, calculated for five time blocks over the 10-min observation period. The duration of block 1 varied among the individual calves depending on induction time, while all of the other blocks were two minutes long. The number of calves in each time block dropped from 13 calves in block 1 to 8 calves in block 5 (*n* = 5 rope, *n* = 3 cuff) because the squeeze was discontinued for some of the calves.

The rate of low-intensity arousals appeared to differ between the application methods for the full 10-min observation period. The number of arousals was somewhat higher and more variable among the individuals in the first block of time that included their induction into a state of reduced responsiveness than in subsequent time blocks (Figure 7), although the two methods did not differ in block 1.

## 4. Discussion

The primary aim of this study was to characterise the responses of healthy newborn calves to a standardised application of the thoracic squeeze. A secondary aim was to evaluate the effect of the method of application on those responses: a soft fabric rope or a modified inflation cuff. The outcomes were compared between the induction categories and between the methods of application. While the magnitude of the difference between the observed medians is large, statistical inferences cannot be drawn due to the small sample size. Therefore, the results were interpreted in terms of confidence intervals and potential clinical significance.

### 4.1. Behavioural Responses

Approximately 80% of the calves were induced into a state of reduced responsiveness, characterized by a loss of posture and a collapse into lateral recumbency, and the cessation of movement. The 13 calves that were successfully induced all did so within 2 min of the squeeze application, regardless of the method of the squeeze. These results are consistent with previous reports in low-vigour calves and foals [3,11,13], as well as healthy foals and piglets [14,24]. In contrast, the remaining three calves were not induced into an apparently reduced state of responsiveness within 3 min of application of the squeeze. These calves did not collapse into lateral recumbency, or they remained in sternal recumbency, showed persistent righting attempts, and eventually made it onto all four feet. This lack of induction may be due to a difference in the application of the squeeze or to some feature of the individual calf itself. There is a possibility that age may have affected the induction success in these calves. However, due to the lack of individual data regarding age of the calves included in the study, there is no evidence to support any age effects. In previous research, healthy foals were subjected to a squeeze at 4 days old, and were successfully induced [24], suggesting that the differences between 12 h and 36 h in the calves of this study likely had no effect on the success of the induction success. More information is needed in order to understand why the squeeze appeared to have no effect on the responsiveness of these calves.

Interestingly, neural reflexes were maintained in almost all of the calves during the period of reduced behavioural responsiveness, with only one calf showing consistently reduced reflexes. In animals under general anaesthesia, the degree to which neural reflexes are reduced is used as an indication of the level of consciousness or awareness [25,26], with the maintenance of reflexes suggesting that the animal is not at a depth which is considered to be fully unconscious [25,26]. This finding in calves is in contrast to the reduction or absence of pedal and palpebral reflexes observed in most healthy piglets subjected to a thoracic squeeze [14]. Moreover, while all of the piglets induced by the squeeze closed their eyes, none of the calves in this study did, even when they showed other induction behaviours such as a loss of posture. Together, these observations suggest that the calves in this study did not reach the same level of reduced responsiveness that was observed in the piglets, but the reasons for this are unknown. This could be due to species differences in the response to the squeeze, or due to differences in the application of the squeeze to animals of different sizes and body morphologies.

Nearly half (seven out of 16) of the induced calves remained in a state of reduced responsiveness for the full 10-min period. Conversely, approximately one third (four out of 13) of the induced calves were later spontaneously aroused, and the squeeze was discontinued. The calves that were fully aroused out of reduced responsiveness did so in the second half of squeeze application (6–8.5 min after the start of the squeeze). It is important to note that the 10-min duration for the squeeze was an arbitrary limit due to a lack of information suggesting a safe squeeze duration for healthy neonates. As a result, it is unknown whether there is a biological difference between the calves that remained in reduced responsiveness for 6 min and the calves that remained induced for the full 10-min duration. However, what is known is that nearly all of the calves were induced into a state of reduced responsiveness for several minutes before self-arousing out of the state or being aroused by a researcher. Whether the observed success of induction and duration for which a state of reduced responsiveness was maintained is typical of calves, healthy or otherwise, is unknown because these are the first detailed data on this phenomenon.

The calves that were spontaneously aroused, for which the squeeze was discontinued, induced faster than the calves that remained in a state of reduced responsiveness for the full 10 min. Furthermore, the calves that later fully aroused showed more low-intensity arousals in the early period following induction and 4–6 min after the application of the squeeze than did the calves that did not spontaneously arouse during the 10-min squeeze. This suggests that the calves for which the squeeze was later discontinued may have been in a shallower state of reduced responsiveness and, as a result, showed more sensitivity to external stimuli than did calves that were fully induced into a state of reduced responsiveness for the full 10 min.

### 4.2. Potential Mechanisms Underlying the Thoracic Squeeze

The mechanisms underlying the responses to the thoracic squeeze, which has now been observed in neonates of four farmed mammal species, are unknown. A number of mechanisms have been proposed to explain the observed responses. Importantly, there is no evidence to suggest that the loss of posture and the cessation of movement observed in the calves and other neonates is due to a fainting episode. Fainting refers to a sudden loss of consciousness caused by a significant decrease in cerebral blood pressure [27]. Typical signs of fainting involve a sudden loss of posture, a change in cardiovascular function (characterised by an increased respiratory rate, a decreased heart rate, decreased blood pressure, and decreased blood oxygen levels) that suggest that blood flow to the brain has been significantly reduced [28,29]. Holdsworth et al. [14] demonstrated, in piglets, that the thoracic squeeze does not cause any clinically marked disturbances in oxygen saturation, heart rate, breathing rate or mucous membrane colour. The two calves for which the squeeze was discontinued due to physiological instability did not show changes consistent with fainting. One calf squeezed with the cuff showed a marked increase in its breathing rate at approximately 5.5 min, and the other showed a noticeable decrease in its heart rate at around the same time. However, all of the other physiological variables which are indicative of cerebral perfusion and blood oxygen level remained stable throughout the squeeze in these calves. In addition, their physiological status remained stable during induction when the loss of posture occurred, and only became unstable some minutes later. As such, there is no evidence to support a suggestion that the physiological changes observed in these two calves were fainting episodes. After the removal of the squeeze, both calves passed all of the recovery criteria within 5 min. However, while this suggests that the thoracic squeeze is generally safe to apply to neonates, the close monitoring of the animals during the squeeze should be recommended.

Another theory which has previously been used to explain the observed responses to a thoracic squeeze relates to the birth process. It has been proposed that a fetus receives a thoracic compression during its passage through the birth canal that, along with neurohormonal factors, facilitates the transition from unconsciousness in utero to consciousness after birth by suppressing consciousness and behavioural responsiveness during birth [12]. It is thought that the thoracic squeeze mimics this suppressive action on behaviour and movement [3,12]. However, this theory has yet to be supported with evidence. Furthermore, based on the proximity to the birth process, it has been suggested that the responses to the thoracic squeeze become extinct as neonates age. Thus, the effects of postnatal age on responses to the thoracic squeeze should be investigated.

Finally, recent research suggests that the observed responses to the squeeze may be attributed to a phenomenon known as tonic immobility (TI) [14]. TI is an immobility state accompanied by reduced responsiveness and analgesia; it has been observed in a wide range of animal species which have been exposed to some form of restraint [30]. This kind of response is apparently elicited by sustained physical contact, and is facilitated by an initial activation of the hypothalamic-pituitary-adrenal (HPA) axis or equivalent, in addition to the increased secretion of glucocorticoids [31]. From there, the response is mediated by specific neural pathways involving brainstem and forebrain structures that cause a range of behavioural and physiological effects that are similar to those observed in neonates during the thoracic squeeze [32,33].

Based on the results of this study, along with previous research [14,24], the observed responses to the thoracic squeeze seem to align most closely with TI. The induction of TI is rapid, and is considered to be partly attributable to a neural reflex [34,35,36]. In the calves, induction always occurred within 2 min of the start of squeeze application and, in most cases, occurred within 1-min. Similarly rapid induction has been observed in piglets [14], foals and lambs [13,24]. TI is typified by behavioural quiescence, characterised by motor inhibition, decreased muscle tone and a loss of posture, as well as changes in heart rate, breathing rate and blood pressure, and an overall reduction in responsiveness to external stimuli [30,37]. Likewise, responses to the squeeze have been reported as being very similar to responses observed during TI, including behavioural and motor quiescence accompanied by decreased muscle tone and an overall reduction in responsiveness to external stimuli in most neonates [14,24].

Finally, induction into TI is apparently dependent on HPA activation, and is associated with changes in pain sensitivity. Thus far, only one study has evaluated HPA responses and pain sensitivity in response to the thoracic squeeze. In healthy neonatal foals, increases in cortisol and adrenocorticotropic hormone (ACTH) concentrations were found during the squeeze, and these were associated with changes in cardiovascular function [24]. Moreover, foals showed reduced behavioural responses to a noxious stimulus applied to the base of the tail, suggesting that the squeeze had an analgesic effect [24]. While these preliminary findings provide support for tonic immobility as the mechanism underlying responses to the thoracic squeeze, the results were from a single study on a single species, with a small sample size (eight foals). Therefore, more research is needed in order to explore whether responses to the thoracic squeeze are equivalent to TI. To further elucidate the link, future research should specifically focus on the HPA activity of calves during the induction and maintenance of the squeeze in order to assess whether cortisol responses follow similar patterns to those of animals in TI.

The role of stress in TI induction may also explain why some individuals are not able to be induced into a state of reduced responsiveness after the application of the thoracic squeeze. The calves in this study that were not induced after the application of the squeeze may not have had a HPA activation that was significant enough to effect a change in responsiveness. There is a need for further research to evaluate the HPA activity of calves that are induced into reduced responsiveness against the HPA activity of non-responders to the squeeze. Determining if responses to the thoracic squeeze are equivalent to TI would inform the potential use of the squeeze as a ‘low-stress’ therapy or handling practice for neonatal farm animals, as has been suggested. In addition, future research should evaluate the pain responses of neonates to noxious stimuli during a thoracic squeeze in order to determine whether the squeeze produces an analgesic effect, and the duration of this analgesia in neonates.

### 4.3. Comparison of the Cuff and Rope Methods

Induction into a state of reduced responsiveness seemed to occur more quickly in calves squeezed with the cuff than in calves squeezed with the rope, with less inter-individual variability in the latency to induction in the cuff group. There was no difference in the number of animals that were spontaneously aroused with each method, and no apparent difference between the methods in the rate of low-intensity arousals while the calves were squeezed.

These findings suggest that both methods of squeeze application were equally effective at inducing and maintaining a state of reduced responsiveness in calves. However, from a practical point of view, the cuff was easier and faster to apply than the rope. The cuff also appeared to provide a more uniform squeeze to the thorax. In contrast, the rope was challenging to apply. Due to friction on the rope, each loop had to be tightened separately, which meant that the force applied to the thorax was different for each loop, and the rope’s application could not be standardized across calves of different sizes and proportions. Because of this, the time taken to apply the rope was longer than that for the cuff, likely resulting in longer latencies to induction. These results are supported by similar findings in healthy piglets, with the inflation cuff being applied faster and eliciting induction more quickly than the rope [14]. In both species, the cuff was quicker to remove from the thorax for calves and piglets that became physiologically unstable during the squeeze, or spontaneously aroused, making the cuff a more practical way to apply a thoracic squeeze to neonates than the rope.

### 4.4. Possible Applications for the Thoracic Squeeze

While more research is needed to characterize the physiological effects of the thoracic squeeze, the results of this study suggest that this technique has potential applications in the veterinary and agricultural industries. The squeeze has been used with reported success as a therapy for low-vigour neonatal foals, calves and lambs showing abnormal behaviours after birth [3,11,13]. In addition, the similarities of responses to the thoracic squeeze and TI suggest that the squeeze may be effective as a non-chemical form of restraint for the purpose of animal handling, or for husbandry purposes on a farm. Another application for the squeeze could be as a form of analgesic for the purpose of the application of painful husbandry procedures, though more research is needed to evaluate the analgesic effects of the squeeze before it can be implemented on a farm.

## 5. Conclusions

The thoracic squeeze elicited a state of reduced behavioural responsiveness in approximately 80% of the calves in this study, characterised by loss of posture and the cessation of movement. Approximately half of the induced calves remained in this state for the full 10-min squeeze period, and all of the induced calves were quiescent for at least 5.5 min. While both application methods were effective, the inflation cuff was faster and simpler to apply and to remove. We therefore recommend the use of an inflation cuff in future research.

These findings align with those previously reported in healthy foals and piglets after the application of a thoracic squeeze, suggesting that this may represent a generalised phenomenon in precocial mammalian neonates. Furthermore, these results demonstrate that the thoracic squeeze is generally safe for inducing and maintaining a state of reduced responsiveness in healthy calves; this provides initial support that the squeeze would be safe as a therapy for low-vigour calves, although close physiological monitoring is recommended throughout the squeeze, and the response may be relatively brief in some individuals. This preliminary study provides a foundation for further research using the inflation cuff to explore the mechanisms underlying the thoracic squeeze, the potential for it to be used in an industry context, and whether the response is equivalent to tonic immobility.

## Figures and Tables

**Table 1:** Behavioural responses and neural reflexes [21,22] recorded during the 10-min thoracic squeeze [14].

Variable	Description
Latency to loss of posture	Time from the start of cuff inflation or rope tightening to enter lateral recumbency from a standing position.
Latency to cessation of movement	Time from the start of cuff inflation or rope tightening to the cessation of all limb and head movements for 3 s.
Pedal reflex	Status of reflex determined by pinching the interdigital cleft between the claws of a front foot to elicit withdrawal of the limb. Present was assigned for a full limb withdrawal response; Reduced for a slight limb withdrawal or a limb twitch response; and Absent for no muscle twitch or limb movement.
Palpebral reflex	Status of reflex determined by lightly brushing or touching the eyelashes or skin in the lateral corner of the eye, to elicit a blink or eye twitch response. Present was assigned for a full twitch or blink response; Reduced for a small twitch response; and Absent for no muscle twitch.
Low-intensity arousals	Low intensity, short duration movements. Characterised by eye opening and vigorous limb and head movements for 10 s or less, before a state of reduced responsiveness was resumed.
High-intensity arousals	High intensity movements, lasting longer than 5 s. Characterised by opening of the eyes and vigorous movements with righting onto all four feet.

**Table 2:** Number and proportion (with 95% confidence intervals) of calves in each induction category, according to the method of squeeze application. See Section 2.6 for a description of the induction categories.

Category	A (Not Induced)	B (Induced but Discontinued)	C (Induced and Maintained)	D (Induced but Unstable)	Total
Rope	2	2	4	0	8
Cuff	1	2	3	2	8
Total	3	4	7	2	16
Percentage (95% CI)	19% (4–46%)	25% (7–52%)	44% (20–70%)	12% (2–38%)	100%

## Data Availability

Contact the corresponding author.

## References

[1] Mellor D., Lentle R.G. (2015). Survival implications of the development of behavioural responsiveness and awareness in different groups of mammalian young. N. Z. Vet. J..

[2] Tuchscherer M., Puppe B., Tuchscherer A., Tiemann U. (2000). Early identification of neonates at risk: Traits of newborn piglets with respect to survival. Theriogenology.

[3] Aleman M., Weich K., Madigan J. (2017). Survey of veterinarians using a novel physical compression squeeze procedure in the man-agement of neonatal maladjustment syndrome in foals. Animals.

[4] Baxter E.M., Jarvis S., D’Eath R.B., Ross D.W., Robson S.K., Farish M., Nevison I.M., Lawrence A.B., Edwards S.A. (2008). Investigating the behavioural and physiological indicators of neonatal survival in pigs. Theriogenology.

[5] Mellor D., Stafford K. (2004). Animal welfare implications of neonatal mortality and morbidity in farm animals. Vet. J..

[6] Laster D.B., Gregory K.E. (1973). Factors Influencing Peri- and Early Postnatal Calf Mortality. J. Anim. Sci..

[7] Raboisson D., Delor F., Cahuzac E., Gendre C., Sans P., Allaire G. (2013). Perinatal, neonatal, and rearing period mortality of dairy calves and replacement heifers in France. J. Dairy Sci..

[8] Barrier A., Ruelle E., Haskell M., Dwyer C. (2011). Effect of a difficult calving on the vigour of the calf, the onset of maternal behaviour, and some behavioural indicators of pain in the dam. Prev. Vet. Med..

[9] Murray C.F., Leslie K.E. (2013). Newborn calf vitality: Risk factors, characteristics, assessment, resulting outcomes and strategies for improvement. Vet. J..

[10] Kolkman I., de Vliegher S., Hoflack G., Van Aert M., Laureyns J., de Kruif A., Opsomer G. (2006). Caesarean section. Slov. Vet. Res..

[11] Stilwell G., Mellor D.J., Holdsworth S.E. (2019). Potential benefit of a thoracic squeeze technique in two newborn calves delivered by caesarean section. N. Z. Vet. J..

[12] Mellor D.J. (2017). Transitions in neuroinhibition and neuroactivation in neurologically mature young at birth, including the potential role of thoracic compression during labour. Aleman, M.; Weich, KM.; Madigan, J.E. Survey of veterinarians using a novel physical compression squeeze procedure in the management of neonatal maladjustment syndrome in foals. Animals.

[13] Flora T., Smallman M., Kutzler M.A. (2021). Resuscitation Compression for Newborn Sheep. Vet. Clin. N. Am. Food Anim. Pract..

[14] Holdsworth S.E., Kells N.J., Chidgey K.L., Vallée E., Ward N., Mellor D.J., Beausoleil N.J. (2021). Characterisation of the Behavioural Effects of a Thoracic Squeeze in Healthy Newborn Piglets. Animals.

[15] Mee J.F. Managing the Calf at Calving Time. Proceedings of the 41st Annual Conference American Association of Bovine Practitioners.

[16] Silva B.T., Henklein A., Marques R.d.S., de Oliveira P.L., Leite S.B.P., Novo S.M.F., Baccili C.C., dos Reis J.F., Gomes V. (2016). Vital parameters of Holstein calves from birth to weaning. Rev. Bras. Med. Vet..

[17] Campler M., Munksgaard L., Jensen M. (2015). The effect of housing on calving behavior and calf vitality in Holstein and Jersey dairy cows. J. Dairy Sci..

[18] Doornbos D.E., Bellows R.A., Burfening P.J., Knapp B.W. (1984). Effects of Dam Age, Prepartum Nutrition and Duration of Labor on Productivity and Postpartum Reproduction in Beef Females. J. Anim. Sci..

[19] Schuenemann G., Nieto I., Bas S., Galvão K., Workman J. (2011). Assessment of calving progress and reference times for obstetric intervention during dystocia in Holstein dairy cows. J. Dairy Sci..

[20] Piccione G., Casella S., Pennisi P., Giannetto C., Costa A., Caola G. (2010). Monitoring of physiological and blood parameters during perinatal and neonatal period in calves. Arq. Bras. Med. Veterinária Zootec..

[21] Cook C., Devine C., Gilbert K., Tavener A., Day A. (1991). Electroencephalograms and electrocardiograms in young bulls following upper cervical vertebrae-to-brisket stunning. N. Z. Vet. J..

[22] Yakan S., Aksoy O., Ermutlu C.S. (2020). Comparison of Use of Isoflurane or Sevoflurane for Anaesthesia Induced by Mask in Calves. Acta Sci. Vet..

[23] RStudio Team (2018). RStudio: Integrated Development for R. RStudio.

[24] Toth B., Aleman M., Brosnan R.J., Dickinson P.J., Conley A., Stanley S.D., Nogradi N., Williams C.D., Madigan J.E. (2012). Evaluation of squeeze-induced somnolence in neonatal foals. Am. J. Vet. Res..

[25] Bradbury A., Clutton R. (2016). Are neuromuscular blocking agents being misused in laboratory pigs?. Br. J. Anaesth..

[26] Verhoeven M.T.W., Gerritzen M.A., Hellebrekers L.J., Kemp B. (2015). Indicators used in livestock to assess unconsciousness after stunning: A review. Animal.

[27] Kapoor W.N. (2000). Syncope. N. Engl. J. Med..

[28] Hainsworth R. (2003). Syncope: What is the trigger?. Br. Med. J. Heart.

[29] Wieling W., Thijs R.D., Van Dijk N., Wilde A.A.M., Benditt D.G., Van Dijk J.G. (2009). Symptoms and signs of syncope: A review of the link between physiology and clinical clues. Brain.

[30] Monassi C.R., Leite-Panissi C.R.A., Menescal-De-Oliveira L. (1999). Ventrolateral periaqueductal gray matter and the control of tonic immobility. Brain Res. Bull..

[31] Ong R.M. (1993). Tonic Immobility by Dogs: A Thesis Presented in Partial Fulfilment of the Requirements for the Degree of Master of Veterinary Science in Veterinary Ethology at Massey University. Ph.D. Thesis.

[32] Brandão M.L., Zanoveli J., Ruiz-Martinez R.C., Oliveira L.C., Landeira-Fernandez J. (2008). Different patterns of freezing behavior organized in the periaqueductal gray of rats: Association with different types of anxiety. Behav. Brain Res..

[33] Terpou B.A., Harricharan S., McKinnon M.C., Frewen P., Jetly R., Lanius R.A. (2019). The effects of trauma on brain and body: A unifying role for the midbrain periaqueductal gray. J. Neurosci. Res..

[34] Gentle M.J., Jones R., Woolley S.C. (1989). Physiological changes during tonic immobility in *Gallus gallus* var *domesticus*. Physiol. Behav..

[35] Valance D., Després G., Richard S., Constantin P., Mignon-Grasteau S., Leman S., Boissy A., Faure J.-M., Leterrier C. (2008). Changes in Heart Rate Variability during a tonic immobility test in quail. Physiol. Behav..

[36] Webster D.G., Lanthorn T.H., Dewsbury D.A., Meyer M.E. (1981). Tonic immobility and the dorsal immobility response in twelve species of muroid rodents. Behav. Neural Biol..

[37] Vieira E.B., Menescal-De-Oliveira L., Leite-Panissi C.R.A. (2011). Functional mapping of the periaqueductal gray matter involved in organizing tonic immobility behavior in guinea pigs. Behav. Brain Res..

